# Occupational exposure limits for psychosocial hazards: A promising concept or a premature leap?

**DOI:** 10.5271/sjweh.4280

**Published:** 2026-07-01

**Authors:** Irina Guseva Canu, Henk F van der Molen

**Affiliations:** 1 Department of Occupational and Environmental Medicine, Center for Primary Care and Public Health (Unisanté); Faculty of Biology and Medicine, University of Lausanne, Lausanne, Switzerland.; 2 Department of Public and Occupational Health Amsterdam UMC, University of Amsterdam, Amsterdam Public Health Research Institute, Amsterdam, The Netherlands.; 3 Societal Participation and Health, Amsterdam Public Health Research Institute, Amsterdam, The Netherlands.

**Keywords:** hazard identification, risk assessment, risk management, occupational mental health

## Abstract

**Objectives:**

We critically examined the proposal to establish occupational exposure limits (OEL) for psychosocial hazards, evaluating its scientific feasibility, methodological challenges, and implications for occupational health practice.

**Methods:**

We reviewed the conceptual framework and recommendations by Pauli et al and compared them with established approaches for chemical and physical hazards. Key obstacles were analyzed, including the reliance on latent constructs, terminological ambiguity, and the absence of objective exposure metrics, while considering the advent of the exposome in epidemiology, emerging technologies and political economy factors.

**Results:**

Our analysis shows that analogies with physical and chemical OEL offer useful insights but cannot be directly applied to psychosocial hazards. Unlike traditional hazards, psychosocial hazards are context-dependent, socially constructed, and often measured through self-reported surveys, limiting the derivation of adverse effect levels. Current psychosocial models of occupational stress aggregate diverse stressors under broad constructs, impeding actionable risk assessment. While organizational hazards such as shift work and long working hours can be objectively quantified using human resource data and sensors, social and moral dimensions remain elusive. Individual biomarkers might not offer adequate diagnostic value, while using multiple biomarkers in combination introduces challenges related to cost and feasibility. Furthermore, regulatory decisions are shaped by economic interests and stakeholder conflicts, complicating consensus and OEL adoption.

**Conclusions:**

A paradigm shift is required: moving from generic theoretical models to specific, measurable indicators, integrating multi-source data, and harmonizing methodologies. Without this transformation, OEL risk over-simplifying complex psychosocial phenomena and failing to achieve meaningful preventive outcomes. For occupational safety and health practice, work organizations should prioritize the more specific identification and measurement of psychosocial hazards, using context-specific data and harmonized methods, to enable more effective risk management and prevention, pending the establishment of formal occupational exposure limits for psychosocial hazards.

In the October 2025 issue of the *Scandinavian Journal of Work, Environment and Health*, Pauli et al (1) advocated for the development of occupational exposure limits (OEL) for psychosocial hazards and derive conceptual and methodological recommendations drawing inspiration from established practices for chemical and physical hazards. Their proposal is timely, given the increasing prevalence of work-related mental health problems (2) and the persistent implementation gap in psychosocial risk (PSR) management across Europe (3). Currently, the strongest scientific evidence links high job demands, effort–reward imbalance, low organizational justice, low social support, high emotional demands, and low decision authority to stress-related mental disorders (4), while job strain, long working hours, effort–reward imbalance, and job insecurity is linked to cardiovascular diseases and mental disorders (5). For other PSR and health outcomes – such as diabetes, obesity, musculoskeletal disorders, pregnancy outcomes, cancer, and digestive diseases – the evidence is weaker or inconsistent (4, 5).

Limited knowledge on how to assess PSR, insufficient expertise and training in PSR, and general regulations lacking in clarity were identified as barriers to PSR assessment and management. In this context, limited knowledge primarily refers to gaps at the scientific and methodological level, particularly in the development and validation of assessment approaches, often in collaboration with companies, rather than to downstream implementation alone. In contrast, traditional occupational hazards are easier to address as they can be inspected using a checklist, a walkthrough of the workplace, or by undertaking instrumental measurements to assess compliance with OEL. When PSR are addressed, they are more limited to the tangible issues such as bullying and harassment complaints rather than risks arising from the organization of work such as job control and job demands (6). And this may be where the essence of the problem hides.

## Challenges in assessing and managing psychosocial risks

In the management of physical and chemical risks, the current trend is to decompose heterogeneous families of chemicals or physical hazards to identify those with adverse effects. The identification of specific uranium compounds with clear dose-dependent carcinogenic and cardiotoxic effects (7, 8) and the demonstration of exacerbated genotoxicity in nanometric titania (9) are two recent examples illustrating the benefit of refining our knowledge to improve hazard identification, the first step toward quantitative risk assessment. Indeed, this led to the reclassification of uranium and titania regarding their carcinogenicity to humans in the International Agency for Research on Cancer (IARC) and European Union frameworks, respectively.

The breakdown of psychosocial hazards, however, remains unchanged. For decades, we have persisted in grouping specific PSR and organizational hazards into latent variables such as *job demands* or *efforts*. Notably, by deconstructing the *efforts* of bus drivers, 39 distinct stressors have been identified and measured (10), enabling actionable interventions. Such specificity is also essential for OEL derivation and application as practical preventative tools.

Conversely, generic psychosocial models of stress, aggregate diverse hazards under broad constructs thereby hindering to provide actionable thresholds (no/low adverse effect level (NOAEL/LOAEL) and OEL), despite well-established dose–response relationships (11). But what can a legislator, labor inspector, or employer do with a critical unitless exposure level of 0.26 for *lack of job control* that integrates 15 different aspects of job control measured in an Australian study (6)? Notwithstanding, this quantitative health-based benchmark dose is now available for *job control* in the PSR assessment and cited among approaches that pave “the way toward OEL” (1). Similarly, adapting the risk matrix approach that uses established dose–response relationships between generic constructs and dichotomized mental health outcomes (12) reinforces the dominance of prevailing models. Like early nanomaterial control banding (13), such a strategy underscores PSR as emerging risks, requiring robust (quasi)experimental and epidemiological studies with precise exposure assessment and contextual specificity.

## Lessons from chemical and physical hazard management

The terminological ambiguity in available surveys on PSR remains another critical issue (14). Stress research still continues to conflate exposure (“stressors” or “risk factor”) with outcomes (“strain” or “risk”), creating circular constructs validated by the same questionnaires that define them (15). In the era of growing exposome integration in epidemiology, we also need valid quantitative measures of exposures and their mixtures to confirm the biological plausibility of their health effects. A latent variable is a theoretical construct that cannot be directly measured. Moreover, each research team constructs them in its own way, often within a unique occupational context, hindering harmonization efforts. The idea to identify the NOAEL and LOAEL of the construct total score that triggers an adverse health effect thus appears questionable.

The analogy with traditional hazards carries many challenges and highlights the risk of over-simplifying PSR assessment by directly transferring toxicological concepts. Although chemical OEL have been applied since the 1940s, procedures for their derivation remain heterogeneous. There is still debate on how to define the critical adverse effect or on endpoint selection. While Pauli et al (1) rightly emphasize the need for harmonization and early detection thresholds which could strengthen primary prevention and shift the focus beyond disease-level outcomes like burnout or depression, a more nuanced consideration of psychosocial hazards would be more precautionary.

## Toward better measurable approaches

Organizational hazards are relatively straightforward to measure quantitatively using human resources (HR) records, legal time-monitoring systems, or payroll data, which are less prone to recall or reporting bias. However, these sources may overlook extra hours that are common but unregulated in some occupations (eg, researchers, physicians, managers) and therefore require cross-checking against self-reported data. Similarly, work ergonomics and some other PSR factors can be assessed through observational tools and sensor-based technologies, like noise, temperature, and potentially airborne chemicals (16). These exposures align more closely with traditional hazards as they are clearly defined and can be quantified and managed through occupational hygiene principles and biosensor use. In contrast, hazards rooted in social or moral dimensions, such as perceived organizational injustice, social support, decision authority, or emotional demands, are inherently subjective and shaped by cultural norms and economic pressures (4). These hazards are context-dependent, socially constructed, and still loosely defined, making their “dose” difficult to measure quantitatively with a sensors or existing routines. Therefore, these hazards have been mostly assessed through self-reported questionnaires, rating individual/subjective perception of exposure and its consequences rather than an instrumental appraisal of exposure intensity and/or frequency.

Furthermore, occupational stress-related disorders are best understood as an interaction issue between the worker and the work environment. They often reflect a normal physiological and psychological response to an abnormal situation, influenced by personal and contextual factors. Therefore, self-reported experiences remain reliable descriptors of burnout as directly asking individuals about their perceived stress levels and symptoms might provide the most accurate insights. While biomarkers in stress research could be useful, they primarily reflect the body’s attempts to restore balance and are influenced by complex systemic interactions, making them insufficient as standalone diagnostic tools. Moreover, to achieve a clinically meaningful estimation of the allostatic load and overload, no less than ten biomarkers should be measured simultaneously (17), raising concerns about cost and acceptability. This makes the definition of the PSR-related critical adverse effects, crucial for OEL derivation, a challenge.

Finally, the political economy of occupational health also plays a significant role in shaping decisions about OEL. As seen in the contested regulation of titania and electromagnetic fields, setting exposure limits is rarely a purely scientific exercise. Even in the context of workers’ compensation for substance-related diseases, individual causality assessments often rely on the probability of causation, requiring careful consideration of statistical and diagnostic uncertainties. For example, national-level adjustments to exposure limits for asbestos and lung cancer have been implemented to address these challenges (18). OEL decisions are inevitably influenced by lobbying, economic interests, and insurance considerations. For psychosocial hazards, where evidence is less tangible and stakeholder interests are more divergent, achieving consensus may be even more difficult. The recognition of burnout as a work-related disease remains limited (19), reflecting the WHO’s classification of burnout as an “occupational phenomenon” rather than a disease. Similarly, conditions such as moral injury have gained recognition primarily through sustained advocacy by specific groups, rather than through unequivocal scientific evidence (20).

## Concluding remarks

We argue that a fundamental paradigm shift is needed to move beyond broad constructs and often self-reported PSR measures toward more specific, measurable, and harmonized approaches before OEL can be effectively established. A key priority is the identification and measurement of concrete psychosocial hazards in the workplace, rather than relying on generic theoretical constructs. This requires clearly distinguishing organizational from individual factors, accounting for concurrent exposures and their potential mixtures, and defining psychosocial hazards in ways that permit objective and reproducible assessment.

Rather than relying solely on a single method, triangulating exposure assessment by integrating multiple data sources—each with their own advantages, limitations, and potential biases—offers a promising strategy to enhance the evaluation of psychosocial hazards and to support preventive measures. In addition to self-reported appraisals, quantitative exposure assessment should increasingly draw on HR records, time-tracking systems, and emerging sensor technologies. To enable comparability and cumulative knowledge building, psychosocial risk factors and related health outcomes must be better identified, defined, and characterized in a harmonized manner, aligned with established hazard identification frameworks—the essential first step in risk assessment and risk management.

Importantly, the establishment of OEL represents only one of several risk management options and is justifiable primarily for hazards whose exposure levels can be monitored using harmonized and validated methods, allowing the effectiveness of occupational safety and health practices to be evaluated. Given the conceptual and practical challenges involved, proactive prevention and effective risk management should remain the central focus, even in the absence of formally established exposure limits.
